# Structures of chloramphenicol acetyltransferase III and *Escherichia coli* β-ketoacylsynthase III co-crystallized with partially hydrolysed acetyl-oxa(dethia)CoA

**DOI:** 10.1107/S2053230X23001206

**Published:** 2023-02-23

**Authors:** Aaron B. Benjamin, Lee M. Stunkard, Jianheng Ling, Jaelen N. Nice, Jeremy R. Lohman

**Affiliations:** a Purdue University, 175 South University Street, West Lafayette, IN 47907, USA; Universidade Nova de Lisboa, Portugal

**Keywords:** ketoacyl synthases, substrate analogs, transferases, chloramphenicol acetyltransferase III, *Escherichia coli* ketoacylsynthase III, acetyl-oxa(dethia)CoA

## Abstract

Acetyl coenzyme A (acetyl-CoA) is a reactive metabolite that nonproductively hydrolyzes in a number of enzyme active sites in the crystallization time frame. In order to elucidate the enzyme–acetyl-CoA interactions leading to catalysis, acetyl-CoA substrate analogs are needed. One possible analog for use in structural studies is acetyl-oxa(dethia)CoA (AcOCoA), in which the thioester S atom of CoA is replaced by an O atom. Here, structures of chloramphenicol acetyltransferase III (CATIII) and *Escherichia coli* ketoacylsynthase III (FabH) from crystals grown in the presence of partially hydrolyzed AcOCoA and the respective nucleophile are presented. Based on the structures, the behavior of AcOCoA differs between the enzymes, with FabH reacting with AcOCoA and CATIII being unreactive. The structure of CATIII reveals insight into the catalytic mechanism, with one active site of the trimer having relatively clear electron density for AcOCoA and chloramphenicol and the other active sites having weaker density for AcOCoA. One FabH structure contains a hydrolyzed AcOCoA product oxa(dethia)CoA (OCoA), while the other FabH structure contains an acyl-enzyme intermediate with OCoA. Together, these structures provide preliminary insight into the use of AcOCoA for enzyme structure–function studies with different nucleophiles.

## Abstract 2

Stable analogs of acetyl-CoA are needed to support structure–function studies of acetyltransferase enzymes. Here, the structures of two enzymes in the presence of an acetyl-CoA analog in which the thioester is replaced by an ester are reported.

## 1. Introduction

**Figure 1:**
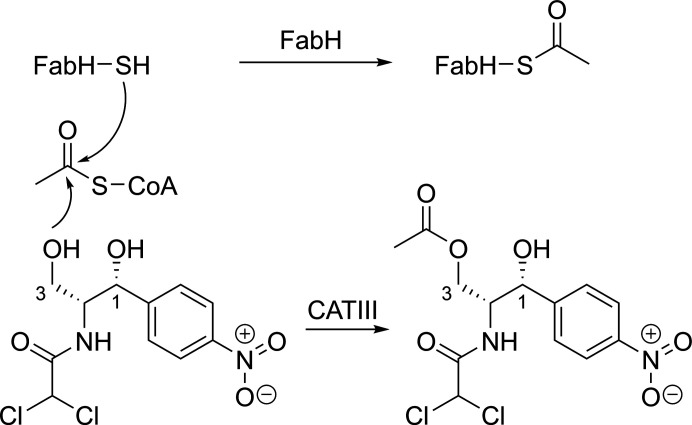
The transthiolation step of FabH and the catalytic activity of CATIII.

Chloramphenicol acetyltransferase III (CATIII) and *Escherichia coli* ketoacylsynthase III (FabH) both transfer an acetyl group from acetyl-CoA to an acceptor: a hydroxyl and a thiol/thiolate, respectively (Fig. 1 ▸). While these enzymes do not share sequence or structural conservation, they both display negative cooperativity in acetyl-CoA substrate binding and catalysis (Ellis *et al.*, 1991 ▸; Alhamadsheh *et al.*, 2007 ▸). The function of negative cooperativity with respect to catalysis is speculative due to a lack of ternary-complex structures. Thus, obtaining structures of these enzymes in complex with substrates would provide insight into the fundamental enzyme–substrate and protein–protein interactions leading to catalysis and cooperativity. The inherent equilibria of the reactions of CATIII and FabH with acetyl-CoA lie so far toward the products that substrate-bound states are difficult to determine. In addition, the inherent reactivity of the acetyl-CoA thioester and nonproductive activation by the enzyme often lead to hydrolysis during crystallization. While there are new methods that may be useful for overcoming the inherent side reactivity of acetyl-CoA during co-crystallization, such as the use of free-electron lasers and serial crystallography, not every system will be amenable as the crystals must survive conformational changes linked to substrate binding (Chapman, 2019 ▸). A tried-and-true method is the use of suitable stable substrate or transition-state analogs.

A substrate analog for both CATIII and FabH is acetyl-oxa(dethia)CoA (AcOCoA), which is expected to be more stable than acetyl-CoA in structure–function studies (Jencks & Gilchrist, 1964 ▸; Yang & Drueckhammer, 2001 ▸). We recently synthesized AcOCoA and similar analogs to support structure–function studies (Stunkard, Kick *et al.*, 2021 ▸; Stunkard, Benjamin *et al.*, 2021 ▸; Stunkard *et al.*, 2019 ▸). However, the synthesis of AcOCoA has previously been reported (Weeks *et al.*, 2018 ▸). In that study, a similar substrate analog, fluoro­acetyl-oxa(dethia)CoA, was hydrolyzed 500-fold more slowly by a thioesterase than the native fluoroacetyl-CoA substrate. The truncated AcOCoA substrate acetyl-oxa(dethia)pante­theine-pivoyl has been crystallized with a thiolase, where it did not participate in the transthiolation or carbon–carbon bond-forming reactions (Meriläinen *et al.*, 2008 ▸). Thus, we initially expected AcOCoA to be relatively stable in CATIII and FabH crystals, both of which crystallize overnight. However, during stability assays we found that FabH was able to hydrolyze AcOCoA to oxa(dethia)CoA (OCoA), albeit extremely slowly compared with acetyl-CoA (Boram *et al.*, 2023 ▸). While improving the synthesis of AcOCoA, we pursued crystal structures of the model enzymes CATIII and FabH using samples of AcOCoA that contained approximately 25–35% OCoA.

CATIII has long been a model enzyme for structure–function studies with relevance to antibiotic drug development (Shaw & Leslie, 1991 ▸). With the recent structure of the ribosome with bound chloramphenicol, there should be renewed interest in finding analogs that retain ribosome inhibition and overcome the activities of CATIII and other chloramphenicol acetyltransferases (Svetlov *et al.*, 2019 ▸). Based on the binding of CoA and chloramphenicol, a hypothesis was developed in which the 1-hydroxyl of chloramphenicol stabilizes a water that acts as a member of the oxyanion hole (Lewendon & Shaw, 1993 ▸; Lewendon *et al.*, 1990 ▸). Thus, some of the catalytic activity and substrate specificity might come from the chloramphenicol substrate itself; in other words, a substrate-assisted catalysis hypothesis. The chloramphenicol analog lacking the 1-hydroxyl is a much poorer substrate, supporting this hypothesis. A ternary structure of AcOCoA and chloramphenicol bound to CATIII could confirm this hypothesis. Furthermore, CATIII is a trimer that displays negative co­operativity with respect to acetyl-CoA. A structure with AcOCoA may reveal conformational differences between the monomers leading to the cooperative behavior. Our structures here reveal a mixture of bound AcOCoA and OCoA, with enough density for AcOCoA to support the substrate-assisted catalysis hypothesis. In addition, each active site of the biologically relevant trimer had varying levels of occupancy. possibly revealing cooperativity.

In *Escherichia coli* and other similar bacteria, FabH carries out the first acyl carrier protein (ACP)-dependent carbon–carbon bond-forming step, making it a target for antibiotic drug development. The two active sites in the FabH homodimer appear to have negative cooperativity in the binding of and/or reaction with acetyl-CoA (Alhamadsheh *et al.*, 2007 ▸). Once one FabH active site has formed the acyl-enzyme intermediate, there is a large decrease in the rate of acetyl­ation of the second active site. Comparing crystal structures in the presence and absence of CoA reveals positional variations in loops that interact between the monomers in the dimer, which may explain some of the negative cooperativity. A convoluting factor is that the active-site loops of FabH can be found in a disordered state (PDB entry 1hnk; Qiu *et al.*, 2001 ▸). The disordered state correlates with our recent finding that FabH displays significant hysteresis when presented with malonyl-CoA as a substrate for decarboxylation in the absence of acetyl-CoA (Boram *et al.*, 2023 ▸). Incubation with acetyl-CoA alleviates the hysteresis, indicating that FabH goes from a disordered state that is not competent for catalysis to a state that is. However, the only structure with an acyl-enzyme intermediate bound has weak density that could be modeled as a partially bound acyl group and an overlapping water (PDB entry 1hnh; electron-density files not deposited; Qiu *et al.*, 2001 ▸). The FabH structures presented here with AcOCoA confirm that the acetyl group is still transferred to generate the acyl-enzyme intermediate. These results reveal that other analogs such as acetyl-aza(dethia)CoA or acetyl-carba(dethia)CoA are needed to capture the acetyl-CoA substrate-bound state of FabH.

The behaviors of CATIII and FabH with AcOCoA provide a comparison of how altering the electrophilic substrate from a thioester to an ester results in very different outcomes with respect to transition-state stabilization. In one case, CATIII, the enzyme is unable to sufficiently stabilize the transition to the product, even on the crystallization time scale of days. With FabH, in contrast, the enzyme is able to generate the thermodynamically unfavorable thioester, albeit with a large excess of substrate, within the same time frame as CATIII. In order to fully comprehend how these enzymes carry out their reactions, it is likely that neutron diffraction will be needed to confirm the positions of H atoms, which are key for catalysis.

## 2. Materials and methods

### 2.1. Macromolecule production

Cloning and protein expression were performed as reported previously for FabH (Boram *et al.*, 2023 ▸). Briefly, *fabH* from *E. coli* K-12 genomic DNA (UniProt P0A5R0) was cloned into a pRSF-derived vector with a TEV protease-cleavable site between the protein and the N-terminal hexahistidine (6×His) tag. We found that the addition of two glycines between the TEV site and the FabH N-terminus was necessary for efficient tag cleavage. The CATIII gene was synthesized by Integrated DNA Technologies according to the sequence found in the transmissible plasmid R387 (UniProt P00484) with appropriate overhangs for Gibson cloning into the modified pRSF vector. Again, double glycines were added to facilitate efficient TEV protease cleavage: CATIII+GG. For CATIII+GG, the primers used were (the additional codons yielding the N-terminal sequence SGGNYTK… are underlined) CATIII+GG-forward, 5′-GAGAACCTCTACTTCCAA *AGTGGTGGTAACTATACAAAATTTGATG*-3′; CATIII+GG-reverse, 5′-CTCGAGGAGATTACGGA*TTATTTTAATTTACTGTTACAC*-3′. Plasmids with *fabH*+GG and *catIII*+GG were transformed into *E. coli* BL21(DE3), which was used as the expression strain. Overnight cultures were used to inoculate LB containing 10 m*M* MgCl_2_, trace metals and 50 µg ml^−1^ kanamycin, which were incubated at 37°C and shaken at 180 rev min^−1^. Upon reaching an OD_600_ of ∼0.5–0.6, the temperature was reduced to 18°C. Once the cultures had reached thermal equilibrium, gene expression was induced by the addition of isopropyl β-d-1-thiogalactopyranoside to a final concentration of 500 µg ml^−1^, with incubation for an additional 16 h. The *E. coli* cells were harvested by centrifugation at 6300 rev min^−1^ and 4°C for 30 min.

*E. coli* cell pellets carrying FabG+GG or CATIII+GG were resuspended in lysis buffer (1 µg ml^−1^ DNase, 1 µg ml^−1^ lysozyme, 300 m*M* NaCl, 20 m*M* imidazole, 10% glycerol, 20 m*M* Tris–HCl pH 8.0), sonicated (60 × 1 s on ice) and clarified by centrifugation at 20 000*g* and 4°C for 30 min. The supernatant was filtered, applied onto a 5 ml HisTrap HP column (GE Healthcare) and washed with lysis buffer using an ÄKTApure fast-performance liquid chromatography system (GE Healthcare). Wash buffer (300 m*M* NaCl, 40 m*M* imidazole, 20 m*M* Tris–HCl pH 8.0) was used to remove additional contaminants, and proteins were eluted with wash buffer containing 500 m*M* imidazole. At this point, the purity of FabG+GG and CATIII+GG from the fractions was analyzed by sodium dodecyl sulfate–polyacrylamide gel electrophoresis (SDS–PAGE). Pure fractions were pooled and cleaved using TEV protease to remove the 6×His tag. FabG+GG and CATIII+GG were then buffer-exchanged into storage buffer (200 m*M* NaCl, 10 m*M* Tris–HCl pH 8.0) followed by the removal of free 6×His tags and 6×His-tagged TEV protease with a 5 ml HisTrap HP column, concentrated and frozen in small aliquots with liquid nitrogen and stored at −80°C. Macromolecule-production information is summarized in Table 1 ▸.

### 2.2. Crystallization

FabG+GG and CATIII+GG were screened against 384 crystallization conditions in 500 nl sitting drops at 20°C set up with a Mosquito (TTP Labtech, Melbourn, UK) to find initial conditions with AcOCoA (partially hydrolysed). FabH+GG at 21 mg ml^−1^ with 10 m*M* AcOCoA produced crystals by the hanging-drop method over 1.0 ml wells containing 1.5% DMSO, 23% PEG 3350, 75 m*M* MgCl_2_, 100 m*M* HEPES–NaOH pH 7.5 or 20% PEG 6000, 3% PEG 400, 100 m*M* MgCl_2_. CATIII+GG at 24 mg ml^−1^ with 10 m*M* AcOCoA and a saturating concentration of chloramphenicol (solid chloram­phenicol was added to the protein and AcOCoA until powder remained in solution followed by centrifugation to precipitate excess material) produced crystals by the hanging-drop method over 1.0 ml wells containing 46% PEG 400, 100 m*M* MgCl_2_, 0.1 *M* sodium citrate pH 5.5 in 4 µl drops (1:3 protein:well solution ratio). Crystals were looped and cooled directly out of the drops with liquid nitrogen. Crystallization information is summarized in Table 2 ▸.

### 2.3. Data collection and processing

X-ray diffraction data for all data sets were collected on LS-CAT beamline 21-ID-G at the Advanced Photon Source at a wavelength of 0.97856 Å. Diffraction intensities were integrated, reduced and scaled using *HKL*-2000 (Otwinowski & Minor, 1997 ▸); data-collection and refinement statistics are listed in Tables 3 ▸ and 4 ▸.

### 2.4. Structure solution and refinement

Molecular replacement with *Phaser* was used to solve the structures of FabH with OCoA (PDB entry 6x7r) or acetylated FabH with OCoA (PDB entry 8d1u) based on the coordinates of PDB entry 1hnj (Qiu *et al.*, 2001 ▸); the structure of CATIII with AcOCoA (PDB entry 6x7q) was solved based on the coordinates of PDB entry 3cla (Leslie, 1990 ▸). Refinement was conducted using *REFMAC* (Murshudov *et al.*, 2011 ▸) in the *CCP*4*i* package (Winn *et al.*, 2011 ▸) with automated model building performed with *ARP*/*wARP* (Langer *et al.*, 2008 ▸) and manual model building with *Coot* (Emsley *et al.*, 2010 ▸).

## 3. Results and discussion

### 3.1. Structure of CATIII in complex with AcOCoA and chloramphenicol

We co-crystallized CATIII in the presence of partially hydrolyzed AcOCoA and chloramphenicol. This produced crystals that grew overnight in various conditions. The vast majority of crystals gave diffraction patterns that were difficult to index due to what appeared to be twinning problems. Serendipitously, we found a single large crystal that diffracted well, was easily indexed in the primitive tetragonal Bravis lattice and was solved in space group *P*4_2_2_1_2 with a trimer in the asymmetric unit. The final structure had good refinement statistics and all residues for each monomer could be modeled. Although no transition metals were added to the crystallization conditions, we found positions for two metals that we modeled as Zn^2+^ based on the *CheckMyMetal* validation server, which would have come from the inclusion of trace metals in the expression medium (Zheng *et al.*, 2017 ▸). The metals are liganded by Glu18 and His22 in each chain and the same residues in a symmetry mate trimer, with the following pattern: chain *A* interacts with symmetry mate chain *C* and chain *B* interacts with symmetry mate chain *B*, creating a larger order hexamer. The previous deposited structures of CATIII belonged to space group *R*32, with a single molecule per asymmetric unit (we would like to note that the numbering scheme of Leslie and Shaw added five residues to 1–74 and six residues to 75–213; here, we use the linear numbering of CATIII; Leslie, 1990 ▸; Leslie *et al.*, 1988 ▸). The *R*32 CATIII structures had two Co^2+^ metal ions bound (0.5 m*M* Co^2+^ was added to the crystallization condition): one site is shared with a metal in our structure that forms the hexamer, while the other Co^2+^ ion occupies a special position situated between the backbone carbonyls of Asn63 and Asp81 with rather long interaction distances of ∼4 Å. While the shared metals generate a larger order hexamer in both crystal forms, all other crystal contacts are essentially unique. Differences in the N-termini of CATIII in previous studies and our study are likely to lead to the variations in crystal packing. The previous *R*32 structures have an N-terminus starting at Met1 and the N-terminal amine has good packing, with a hydrogen bond to the carbonyl of Lys211 that is likely to stabilize the crystals and is not possible without a free amine. Our construct has an N-terminus with a Ser-Gly-Gly-Asn2 cloning artifact that would disrupt the packing seen in the *R*32 structures. The *R*32 crystal packing was reported to deteriorate upon exposure to CoA, preventing elucidation of the binding of acetyl-CoA or analogs via soaking (Leslie *et al.*, 1986 ▸). Thus, our structure provides insight into engineering crystal contacts that allow the production of trimers in the asymmetric unit in order to understand the subtle conformational changes associated with negative cooperativity.

**Figure 2:**
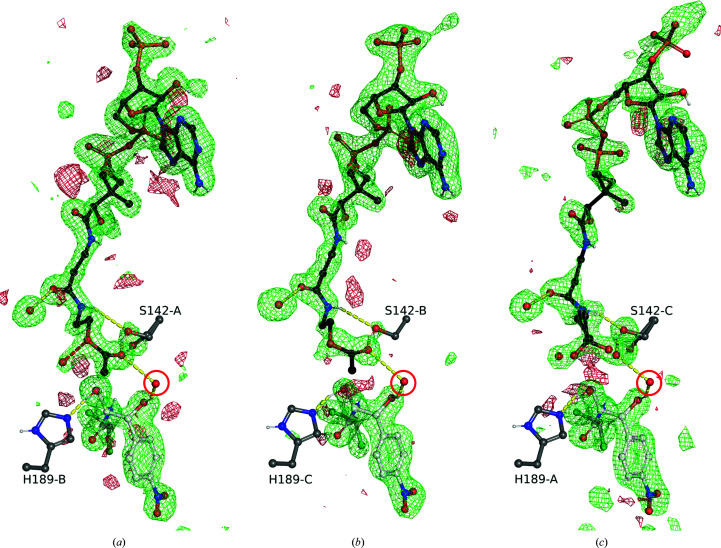
Electron density for CATIII ligands in the active sites in molecules *A* (*a*), *B* (*b*) and *C* (*c*) in the trimer. AcOCoA or OCoA is shown as black sticks, chloramphenicol is shown as white sticks and CATIII active-site residues are labeled and shown as gray sticks. The water participating in the oxyanion hole and hydrogen-bonded to chloramphenicol is circled in red. The σ_A_-weighted *mF*_o_ − *DF*_c_ maps for omitted ligands are shown at +3σ in green and −3σ in red as 5 Å bricks. The Ser142 side-chain hydroxyl was also omitted in order to judge occupancy. Note that a water molecule close to the ester O atom in (*a*) lies in a similar position as a partially occupied water in (*c*), revealing some presence of OCoA in (*a*).

Our CATIII structure has clear electron density for chloram­phenicol in each monomer, which resides in exactly the same orientation as in previous structures. The electron density for AcOCoA/OCoA is relatively clear in chain *A*, somewhat clear in chain *B* and difficult to model in chain *C* (Fig. 2 ▸). We take the differences in electron density to correspond to negative cooperativity, but differences in crystal packing cannot be ruled out. The AcOCoA in chain *A* participates in crystal packing and is not free to leave, while the AcOCoA molecules in chains *B* and *C* are open to solvent. A comparison of the chains shows slight differences in the loops surrounding the CoA binding pocket in chain *C*, further supporting the idea of our electron density reflecting negative cooperativity.

**Figure 3:**
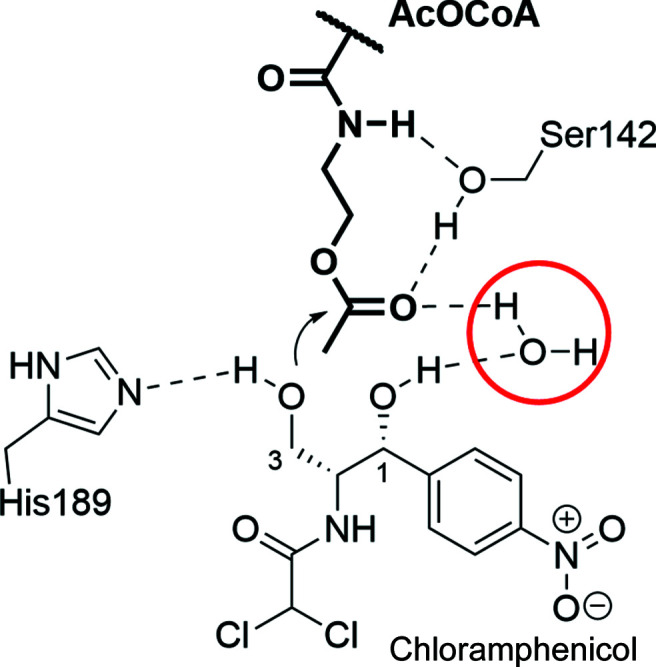
Active-site interactions of AcOCoA with CATIII and chloramphenicol co-substrate. Note that a water bound to chloramphenicol helps to create the oxyanion hole, which is circled in red.

In monomer *A* we can clearly model the position of the acetyl group, even though the electron density supports about 20% OCoA, in which case a water molecule takes the place of the acetyl ketone O atom (Fig. 2 ▸). The binding of the acetyl group is almost exactly as predicted by Leslie and Shaw based on a structure with coenzyme A bound (not deposited in the PDB; Fig. 3 ▸; Shaw & Leslie, 1991 ▸; Leslie *et al.*, 1988 ▸). The location of a water bound between the acetyl ketone and the 1-hydroxyl of chloramphenicol suggests that it stabilizes the transition state in concert with His189. This is the first time that a substrate analog of acetyl-CoA has been found in the CATIII active site, revealing that an active-site water interacts with the tetrahedral intermediate, confirming the substrate-assisted catalysis hypothesis. Nevertheless, the positions of the H atoms that are key to catalysis remain to be determined. Our preliminary structures here provide a platform upon which to perform neutron diffraction to obtain a clear idea of how the transition state is set up.

### 3.2. Structures of FabH in complex with OCoA

We co-crystallized FabH in the presence of partially hydrolyzed AcOCoA. This produced crystals that grew overnight in various conditions. The crystals grown from PEG and Mg^2+^ diffracted well, and were indexed in the primitive tetragonal Bravis lattice and solved in space group *P*4_1_2_1_2 (*a* = *b* = 73 Å) with a monomer in the asymmetric unit (Table 1 ▸). The final structures had good refinement statistics and all residues for each monomer could be modeled. The previous structures of *E. coli* FabH fall into one of three crystal forms. Crystal type I (PDB entries 1hnd, 1hnh, 1hnj and 1hnk; Qiu *et al.*, 2001 ▸) is identical to that reported here, while the others are primitive orthorhombic. Crystal type II belongs to space group *P*2_1_2_1_2_1_, with unit-cell parameters *a* = ∼63, *b* = ∼65, *c* = ∼163 Å (PDB entries 1hn9, 3il9, 5bns and 1ebl; Qiu *et al.*, 2001 ▸; McKinney *et al.*, 2016 ▸; Gajiwala *et al.*, 2009 ▸; Davies *et al.*, 2000 ▸), and crystal type III belongs to space group *P*2_1_2_1_2_1_, with unit-cell parameters *a* = ∼64, *b* = ∼81, *c* = ∼122 Å (PDB entries 2eft, 2gyo, 5bnm and 4z8d; Alhamadsheh *et al.*, 2007 ▸; McKinney *et al.*, 2016 ▸), with both having a dimer in the asymmetric unit. Solid density for CoA (PDB entries 1hnd and 1hnj) and partial density for the acyl-enzyme intermediate (PDB 1hnh) have only been seen in crystal type I (Qiu *et al.*, 2001 ▸), with weak CoA density in crystal type II (PDB entry 1ebl; Gajiwala *et al.*, 2009 ▸) and crystal type III (PDB entries 2eft and 2gyo; Alhamadsheh *et al.*, 2007 ▸). Based on structural alignments between crystals of type I and types II and III, it appears that an N-terminal His tag may be responsible for favoring type II/III crystals due to clashes that would be present in type I. In our crystals, we observe some disorder in the Ser-Gly-Gly-Met1 tag artifact; however, there is sufficient room that the tag artifact still allows the tight crystal packing found in crystal form I. Elongating the tag may be a way to favor the production of type II/III crystals, which would be helpful for capturing the differences between the active sites associated with negative cooperativity.

**Figure 4:**
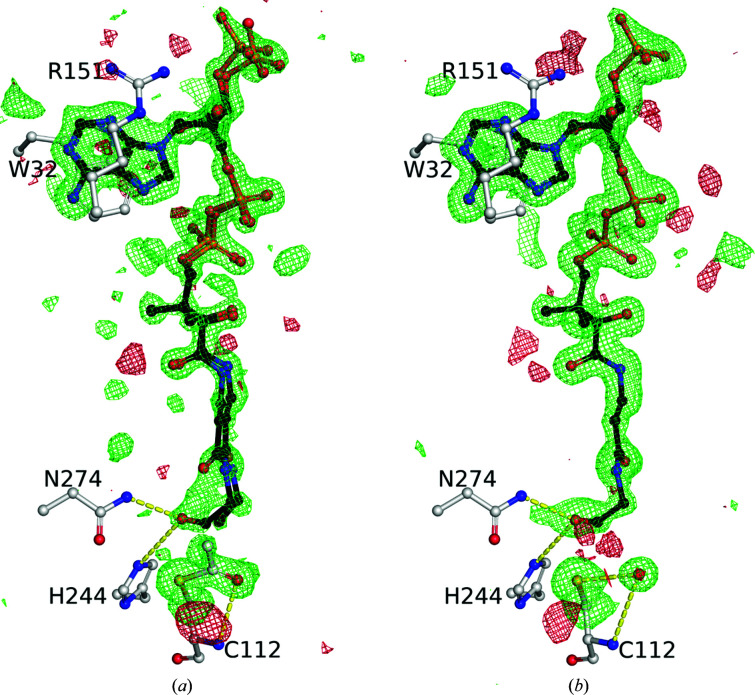
Electron density for FabH ligands. OCoA is shown as black sticks and labeled FabH active-site residues as white sticks. The σ_A_-weighted *mF*_o_ − *DF*_c_ maps are shown at +3σ in green and −3σ in red as 5 Å bricks for (*a*) omitted OCoA, Cys112 C^β^ and sulfur or (*b*) Cys112 C^β^, sulfur and acetyl group. (*a*) is Ac-FabH + OCoA and (*b*) is FabH + OCoA. Note that a water molecule takes the place of the acetyl-cysteine carbonyl.

Slight differences in the crystallization conditions for our structures resulted in differing amounts of density for the acyl-enzyme intermediate and the OCoA product (Fig. 4 ▸). The *B* factors for the C atoms of the acetyl group of the acyl-enzyme intermediate are ∼1.5–2 times as large as those for the atoms of the cysteine to which they are attached, suggesting maybe 50% occupancy. Our structure with electron density for the acyl-enzyme intermediate has relatively poor electron density for the bound OCoA. Our structure with excellent density for OCoA has no density for the acyl-enzyme intermediate. Taken together, we can obtain a clear view of how the enzyme interacts with the products of AcOCoA. The acyl-enzyme intermediate is very similar in structure to that previously determined with acetyl-CoA (PDB entry 1hnh; Qiu *et al.*, 2001 ▸). Similarly, our structure with clear OCoA bound is almost identical to that with CoA bound (PDB entries 1hnd and 1hnj).

**Figure 5:**
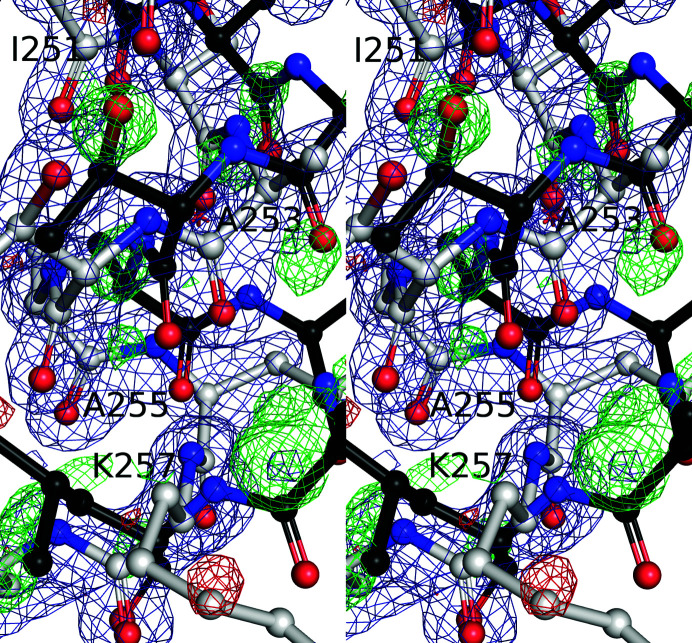
Our FabH structure has alternative conformations for residues 249–258 at low occupancy. Our refined Ac-FabH + OCoA model is shown as white sticks with every other residue labeled, while the residues of FabH in PDB entry 1ebl chain *A* are shown in black. The Ac-FabH + OCoA σ_A_-weighted 2*mF*_o_ − *DF*_c_ map is shown in blue and *mF*_o_ − *DF*_c_ maps are shown at +3σ in green and −3σ in red. Note how the residues for FabH in PDB entry 1ebl occupy the residual green positive density in our maps, suggesting that some fraction of the alternative conformation is populated in our crystals.

Unfortunately, due to the presence of only a monomer in our asymmetric unit, it is difficult to gain insight into the negative cooperativity displayed by the enzyme. Nevertheless, careful inspection of the electron-density maps and comparisons with other structures reveals conformational heterogeneity that might help to explain some of the cooperative behavior. The α-helix between Leu249 and Leu258 has spurious density, suggesting that the helix is in an alternate conformation part of the time. Alignment of our FabH structures with the FabH structures in PDB entries 3il9 (Gajiwala *et al.*, 2009 ▸) and 1ebl (Davies *et al.*, 2000 ▸) reveals the secondary conformation of this loop, likely reflecting a state without CoA bound (Fig. 5 ▸). Questions remain concerning the transthiolation reaction: is the active-site cysteine in the thiol or thiolate state, and how does the protonation state of His244 change before and after the formation of the acyl-enzyme intermediate? The multiple water and nearby side-chain conformations suggest that we do not have the data to accurately interpret how the acyl-enzyme intermediate alters these protonation states. We expect that substrate analogs such as acetyl-aza(dethia)CoA or acetyl-carba(dethia)CoA are needed to capture a ‘substrate-bound’ state of FabH and to overcome the conformational heterogeneity found in these FabH structures.

### 3.3. Different behaviors of AcOCoA in CATIII/FabH active sites

CATIII does not perform the forward reaction between AcOCoA and chloramphenicol during the crystallization time frame at pH 5.5. Δ*G* for the reaction is expected to be 0, which should give us an equal amount of substrate and product. In our case we used a large excess of chloramphenicol, which should have driven the reaction forward, leaving only OCoA. There are a couple of explanations for the lack of acyl-transfer in this context. (i) The Δ*G* ^‡^ is inherently too large for trans­esterification to be efficiently overcome by the enzyme due to geometric or electronic properties of the ester compared with the thioester. (ii) The crystallographic pH is far enough below the p*K*_a_ of the active-site histidine (6.3) to inhibit the reaction (Shaw & Leslie, 1991 ▸). We expect that follow-up studies examining the reaction at various pH values may shed light on which of these two hypotheses is correct.

FabH does hydrolyze AcOCoA very slowly under the relatively dilute conditions used in enzymology experiments compared with the crystallographic conditions used here, where AcOCoA is only in an approximately tenfold excess; as such, the rate of FabH hydrolysis is a concern (Boram *et al.*, 2023 ▸). Our structural studies here confirm that hydrolysis can occur through the acyl-enzyme intermediate. The formation of the acyl-enzyme intermediate is somewhat unexpected, as it is unfavorable with a positive Δ*G*. We used approximately tenfold more AcOCoA than FabH in our crystallization conditions, which may have contributed to the spontaneous formation of the acyl-enzyme intermediate. We observed a similar rate of hydrolysis for AcOCoA and acetyl-CoA in the absence of a malonyl-thioester substrate at pH 8, which is ten times higher than background hydrolysis in buffer, suggesting that FabH does have a noticeable effect (Boram *et al.*, 2023 ▸). However, it did not appear that AcOCoA acts as an acyl donor, since the reaction in the presence of malonyl-CoA did not yield more product than malonyl-CoA alone. Similar to CATIII, it appears that it is geometric or electronic considerations that become rate-limiting in the reaction of FabH with AcOCoA. Future studies examining substrate binding of acetyl-CoA will require a different strategy to using AcOCoA, such as more stable analogs.

Our structures demonstrate different behaviors of AcOCoA depending on the context. AcOCoA is a substrate analog for CATIII but a very slow substrate for FabH. It may be the case that hydroxyl acceptors in general will lead to substrate-analog behavior and thiol acceptors will lead to product formation. With the aforementioned fluoroacetyl-CoA hydrolase, which generates a threonine-based acyl-enzyme intermediate, fluoroacetyl-oxy(dethia)CoA was a substrate with a 500-fold slower rate (Dias *et al.*, 2010 ▸; Weeks *et al.*, 2010 ▸, 2018 ▸). Together, this suggests that acyl-oxy(dethia)CoAs are likely to be useful tools for studying many enzymes with hydroxyl nucleophiles. The catalytic interactions of many Gcn5-related *N*-acetyltransferases (GNATs) still remain uncharacterized due to the spontaneous hydrolysis of acetyl-CoA and reactivity with their substrates. Our studies here with CATIII suggest that acyl-oxy(dethia)CoAs might be effective in capturing the ternary complexes; however, their reactivity with amine nucleophiles remains to be examined.

## Supplementary Material

PDB reference: CATIII, complex with chloramphenicol and acetyl-oxa(dethia)CoA, 6x7q

PDB reference: FabH, complex with oxa(dethia)CoA, 6x7r

PDB reference: FabH with an acetylated cysteine, complex with oxa(dethia)CoA, 8d1u

## Figures and Tables

**Table 1:** Macromolecule-production information

Protein	FabH	CATIII
Source organism	*E. coli* K12	Synthetic gene based on plasmid R387
Expression vector	pRSF	pRSF
Expression host	*E. coli* BL21(DE3)	*E. coli* BL21(DE3)
Complete amino acid-sequence of the construct produced†	MGSSHHHHHHSGSENLYFQ↓SGGMYTKIIGTGSYLPEQVRTNADLEKMVDTSDEWIVTRTGIRERHIAAPNETVSTMGFEAATRAIEMAGIEKDQIGLIVVATTSATHAFPSAACQIQSMLGIKGCPAFDVAAACAGFTYALSVADQYVKSGAVKYALVVGSDVLARTCDPTDRGTIIIFGDGAGAAVLAASEEPGIISTHLHADGSYGELLTLPNADRVNPENSIHLTMAGNEVFKVAVTELAHIVDETLAANNLDRSQLDWLVPHQANLRIISATAKKLGMSMDNVVVTLDRHGNTSAASVPCALDEAVRDGRIKPGQLVLLEAFGGGFTWGSALVRF	MGSSHHHHHHSGSENLYFQ↓SGGNYTKFDVKNWVRREHFEFYRHRLPCGFSLTSKIDITTLKKSLDDSAYKFYPVMIYLIAQAVNQFDELRMAIKDDELIVWDSVDPQFTVFHQETETFSALSCPYSSDIDQFMVNYLSVMERYKSDTKLFPQGVTPENHLNISALPWVNFDSFNLNVANFTDYFAPIITMAKYQQEGDRLLLPLSVQVHHAVCDGFHVARFINRLQELCNSKLK

† ↓ indicates the TEV protease cleavage site.

**Table 2:** Crystallization

	Ac-FabH + OCoA	FabH + OCoA	CATIII + AcOCoA
Method	Vapor diffusion, hanging drop	Vapor diffusion, hanging drop	Vapor diffusion, hanging drop
Plate type	VDX	VDX	VDX
Temperature (K)	298	298	298
Protein concentration (µ*M*)	680	680	960
Buffer composition of protein solution	10 m*M* AcOCoA, 200 m*M* NaCl, 10 m*M* Tris–HCl pH 8.0	10 m*M* AcOCoA, 200 m*M* NaCl, 10 m*M* Tris–HCl pH 8.0	10 m*M* AcOCoA, saturated chloramphenicol, 200 m*M* NaCl, 10 m*M* Tris–HCl pH 8.0
Composition of reservoir solution	75 m*M* MgCl_2_, 20% PEG 6000, 3% PEG 400	1.5% DMSO, 75 m*M* MgCl_2_, 23% PEG 3350, 100 m*M* HEPES–NaOH pH 7.5	100 m*M* MgCl_2_, 46% PEG 400, 100 m*M* sodium citrate pH 5.5
Volume and ratio of drop	4 µl; 2:2 protein:well solution	4 µl; 2:2 protein:well solution	4 µl; 1:3 protein:well solution
Volume of reservoir (ml)	1.0	1.0	1.0

**Table 3:** Data collection and processing Values in parentheses are for the outer shell.

	Ac-FabH + OCoA	FabH + OCoA	CATIII + AcOCoA
Diffraction source	APS beamline 21-ID-G	APS beamline 21-ID-G	APS beamline 21-ID-G
Wavelength (Å)	0.97856	0.97856	0.97856
Temperature (K)	80	80	80
Space group	*P*4_1_2_1_2	*P*4_1_2_1_2	*P*4_2_2_1_2
*a*, *b*, *c* (Å)	72.63, 72.63, 102.87	72.63, 72.63, 102.87	106.92, 106.92, 126.60
α, β, γ (°)	90, 90, 90	90, 90, 90	90, 90, 90
Resolution range (Å)	50–1.30 (1.35–1.30)	50.0–1.35 (1.40–1.35)	50.0–1.68 (1.74–1.68)
Total No. of reflections	247744	224532	311296
No. of unique reflections	68034	60608	84122
Completeness (%)	99.6 (96.5)	98.7 (100)	99.8 (100)
Multiplicity	14.2 (12.0)	13.9 (13.7)	14.5 (14.3)
〈*I*/σ(*I*)〉	34.4 (1.94)	25.1 (3.85)	23.5 (2.97)
*R*_r.i.m._	0.070 (0.568)	0.089 (0.797)	0.152 (1.308)
*R*_p.i.m._	0.019	0.024	0.040
Overall *B* factor from Wilson plot (Å^2^)	14.74	20.81	20.37

**Table 4:** Structure solution and refinement Values in parentheses are for the outer shell.

	Ac-FabH + OCoA	FabH + OCoA	CATIII + AcCoA
Resolution range (Å)	22.99–1.30 (1.35–1.30)	23.59–1.35 (1.38–1.35)	34.33–1.68 (1.72–1.68)
Completeness (%)	99.6	98.7	99.8
No. of reflections, working set	67957	57495	80050
No. of reflections, test set	3446	3041	4005
Final *R*_cryst_	0.144	0.145	0.157
Final *R*_free_	0.167	0.176	0.201
Cruickshank DPI	0.0458	0.0500	0.0933
No. of non-H atoms			
Protein	2358	2355	5284
Ion	1	1	2
Ligand	49	52	230
Solvent	457	413	809
Total	2865	2821	6329
R.m.s. deviations			
Bond lengths (Å)	0.015	0.012	0.012
Angles (°)	2.091	1.909	1.783
Average *B* factors (Å^2^)			
Protein	14.1	22.0	21.4
Ion	32.8	35.5	16.3
Ligand	22.8	26.1	34.4
Water	29.7	34.5	32.7
Ramachandran plot			
Most favored (%)	98	97	100
Allowed (%)	2	3	0
